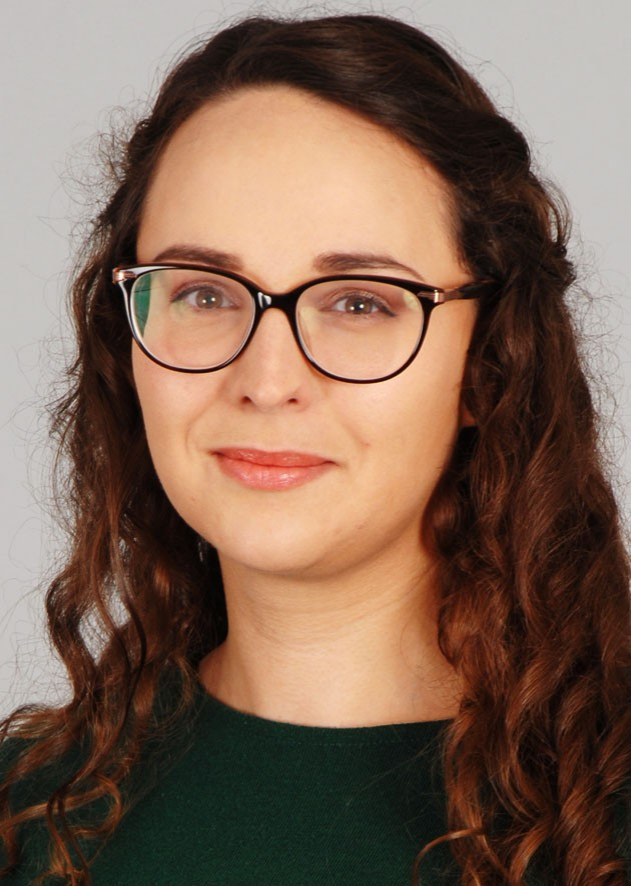# Nothing About Us Without Us – Towards Genuine Inclusion of Disabled Scientists and Science Students Post Pandemic

**DOI:** 10.1002/chem.202100268

**Published:** 2021-06-08

**Authors:** Julia P. Sarju

**Affiliations:** ^1^ Department of Chemistry University of York Heslington YO10 5DD UK

**Keywords:** Covid-19 pandemic, equality, disability, diversity, inclusion

## Abstract

Scientists and students with disabilities have been severely affected by the COVID‐19 pandemic, and this must be urgently addressed to avoid further entrenching existing inequalities. The need for rapid decision‐making, often by senior colleagues without lived experience of disabilities, can lead to policies which discriminate against scientists with disabilities.

This article reflects on disability declaration statistics and research in critical disability studies and social science to explore the challenges experienced by disabled scientists before and during the COVID‐19 pandemic and highlights recommendations and examples of good practice to adopt in order to challenge ableism in STEM communities and workplaces. It is vital that disabled staff and students are fully involved in decision making. This is particularly important as we continue to respond to the challenges and opportunities associated with the ongoing COVID‐19 pandemic and plan for a post‐COVID‐19 future.

This time of great change can be used as an opportunity to listen, learn, and improve working conditions and access for scientists with disabilities, and by doing so, for everyone.

## Graphical abstract

**Mind the gap**! Disabled scientists and students have been severely affected by the COVID‐19 pandemic; this must be urgently addressed to avoid entrenching existing inequalities. The graphical abstract includes a bar chart comparing the % disability declaration statistics for the UK population (∼20% 2015–16), STEM first degree students (15.5%, 2018–19), STEM academics (3.8%, 2018–19), and UKRI grant applicants (∼1%, 2014–19); and highlights the key recommendations given in the article: meaningful consultation, flexible working policies, disabled staff networks, and allyship.

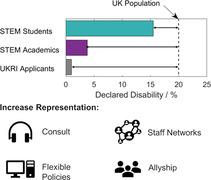

## Lived Experience of Disability Enriches Science

I would like to begin by sharing a story about one of my personal science heroes, a scientist whose talent and lived experience of disability has enhanced her research, her field, and beyond. This scientist is Dr. Wanda Diaz‐Merced. Until 2019, she led the AstroSense project at the South African observatory‘s Office of Astronomy for Development (OAD), and now is expanding on her equity, inclusion, and accessibility work at the International Astronomical Union (IAU) Office for Astronomy Outreach in Japan.

When Dr. Diaz‐Merced began losing her sight during her teenage years, she worried about whether she would be able to study physics.^[1]^ Later, while at university, a friend played her an audio recording of a sunburst, this introduced the possibility of using solar soundscapes to analyse astronomical data as an alternative to visualisations. She developed sonification software with Robert Candey at NASA Goddard Spaceflight Centre, which she went on to verify during her PhD research under the supervision of Professor Stephen Brewster.[^2^, ^3^]

When asked recently about her personal hopes for the future, Dr. Diaz‐Merced answered, *“I would love to be a working astronomer, carrying out research. But right now I don't see any opportunity, so I have to look at the bigger picture. When my contract runs out on 31 December, I'm going home to Puerto Rico, where I hope I can teach. I want a field where we all work as equals, and where factors such as age, disability, gender and socio‐economic status do not control my progress. I want my field not to underuse, misuse or neglect the human potential we all have for exploration and inquiry, and to trust that we all may contribute just as we are.”* ^[4]^

Dr. Diaz‐Merced has contributed significantly to science, she has not “overcome disability”, her lived experience of disability has been integral to her work. However, she, like many other minoritized scientists, cannot see a place for herself in academia. Being inclusive of disabled scientists is not about being charitable, or simply meeting a legal obligation; lived experience of disability enriches science and the scientific community. This article is focussed on the experiences of disabled scientists but exclusionary and discriminatory practices are endemic in society. The public should be able to see scientists who represent them and share their values and experiences. Representation does not happen by accident but it leads to further inclusion.

## Disability: Hidden and Excluded Diversity

It is important to note that the individual experiences of disabled scientists are diverse and intersecting with other identities, an individual may face multiple biases simultaneously.[^5^, ^6^, ^7^] Experiences of disability are broad and include chronic conditions, mental ill‐health, and neurodivergence such as autism, ADHD, and specific learning differences in addition to physical disabilities. The majority of disabilities are invisible and many conditions fluctuate.^[8]^ The nature of the disability, whether it is visible or invisible, and fluctuation of “symptoms” affect how likely an individual is to declare a disability.

### How do we define disability?

Legal definitions mostly focus on an individual's impairment or perceived impairment. For example, the UN Convention on the Rights of Persons with Disabilities states that “*persons with disabilities include those who have long‐term physical, mental, intellectual or sensory impairments which in interaction with various barriers may hinder their full and effective participation in society on an equal basis with others*”.^[9]^ Many disability scholars and activists find it more productive to consider the Social Model of Disability which focusses on disabling barriers, ableism, and societal/attitudinal issues rather than considering disability to be a medically inevitable consequence of an impairment.[^10^, ^11^, ^12^]

Ableism is discrimination against a person or people on the basis of disability and *‘within ableism the existence of disability is tolerated rather than celebrated as a part of human diversification’*.^[13]^

### Language matters

Which language to use when discussing disability is “*hotly debated*” and there are a wide range of views.^[14]^ Personally, I view myself as somewhere between disabled and ally, varying with insecurities and fluctuating severity of symptoms. I feel most comfortable, or if I am being completely honest, least uncomfortable with the language “lived experience of disability” when describing myself. Ultimately, we need to be sensitive to and respect each individual person's choice to describe themselves and their experience/s.

### Declaration of disability

Let's take a quick look at the numbers. Disabled people are a sizable minority, with 1 in 5 people in the UK and 1 in 8 people in the US identifying as having a disability.^[15]^ Can any of us say that anywhere near 1 in 5 of the people we work with are openly disabled, or 1 in 5 of our institutional leaders? I say ‘openly’ disabled because there will be a hidden diversity of individuals who do not declare their disability status. Reviewing the statistics for participation in academia and STEM careers for individuals declaring disabilities, we can see that the rates of known disability are lower compared to general population.[^16^, ^17^, ^18^] Discrimination, insufficient accessibility,^[19]^ and attitudinal issues all contribute to both under‐representation of disabled scientists and declaration of disability status. Underreporting of disability status is linked to fears of being treated differently and discriminated against,[^14^, ^20^] and internalised ableism (where negative attitudes towards disability affect how individuals perceive themselves and how comfortable they feel identifying as disabled).[^13^, ^16^]

There are at least two major issues here. The first is that there is a large number of scientists who do not feel they can be their whole selves in their workplaces, are not accessing accommodations or support, and may have to moderate what they say and how they act to “pass”. The second is that disabled people do not have equitable access to scientific careers. Following a recent major survey of its membership,^[21]^ the Royal Society of Chemistry concluded that “*across all of our activities, people with self‐reported disabilities are under‐represented compared with the national average*” and this is consistent with the National Science Foundation in the US who “*identified persons with disabilities as among those who are under‐represented in science and engineering education and employment”*.^[17]^ Disabled scientists face physical and attitudinal barriers and of course, these issues extend beyond scientific careers. In the UK, the employment rate for disabled people (aged 16 to 64 years) was 53.2 % in 2019, compared with 81.8 % for non‐disabled people.^[22]^ Delving into the UK employment data in more detail, the employment rate was highest (between 59.0–59.8 %) for those with physical disabilities affecting the legs, feet, back or neck and lowest (17.6 %) for disabled people with severe or specific learning difficulties. Individuals reporting mental illness had the second‐lowest employment rate (28.5 %).

### Disability in academia

In 2018–2019, 15.5 % of first‐degree STEM students studying in the UK declared at least one disability, and analysis of the destinations of STEM graduates found that STEM graduates with a known disability are more likely to be unemployed six months after graduation compared to those STEM leavers with no known disability.^[18]^ Furthermore, this study found that STEM first degree and postgraduate leavers with a social communication/autistic spectrum disorder have the highest rate of unemployment six months after leaving and the lowest percentage going into professional employment in 2016/2017.

Science graduates with disabilities should be able to pursue further study and a scientific career if they wish, but is this the case? A recent study by the Careers Research & Advisory Centre commissioned by the Royal Society reported that declaration is lower in STEM disciplines than outside STEM.^[23]^ Perhaps unsurprisingly, declaration rates also varies dramatically with career stage with the lowest rates reported amongst senior staff and the highest rates amongst first degree students. However, there is some reason for optimism as the percentage of STEM academic staff with a declared disability is rising, with an increase from 2.0 % in 2007/2008 to 3.8 % in 2018/2019.^[18]^

This report also highlighted specific barriers to success and progression identified by STEM academics including competitive research environments where there are expectations of consistent high levels of research productivity (see example participant quote below), difficulties in obtaining external research funding, expectations of full‐time working patterns, and challenges accessing necessary adjustments. Importantly, the specific challenges and exclusionary practices experienced by disabled scientists were diverse and varied widely, impacted by individual circumstances, the nature of disability, and intersection with other protected characteristics such as gender, race, and sexuality.

“*I simply cannot work the long hours that I did before I was unwell. I have ongoing fatigue alongside heart issues, some (thankfully fairly minor) memory problems, and compromised immunity. This makes it extremely challenging to be as productive as I used to be. I am certain that this will affect my ability to publish and “keep up” with my contemporaries, and so I suspect that this will affect how I am compared to others when applying for a permanent academic role and grants*.”

(Female, ECR, Chemistry, Physical disability or health condition)^[23]^

A key issue for scientists in academia is attracting research funding and the additional challenges for disabled researchers has been recently highlighted by the case of Professor Justin Yerbury. After a successful appeal by Professor Justin Yerbury, Australia's National Health and Medical Research Council have updated their practices to make it clear that the impact of disability on the applicant's research output should be considered by peer reviewers.^[24]^ This should be adopted by all funding bodies. Recent analysis by the major UK‐based funding body, UKRI, reported that only 1 % of applicants for research funding disclose that they have a disability and that principal investigator (PI) applicants with a declared disability have lower award rates than those who said they do not have a disability (the number of PI and Fellow awardees declaring a disability was too low for statistical analysis).^[25]^ One of UKRI “ambitions” in response to this report is to increase declaration rates, but how will this information be used? In a recent blogpost, the new CEO of UKRI, Professor Dame Ottoline Leyser, outlined her vision for a more equitable future, requiring systematic change, with training and community at the heart.^[26]^

Considering the data showing a lack or in many cases a complete absence of senior staff and studies of disabled scientists reporting the importance of visible role‐models, it should be a priority to develop and encourage disabled leaders. The barriers highlighted for developing disabled leaders in the higher education sector are just as relevant disabled scientists in industry and universities, they include limitations of laws to prevent discrimination, stereotyping, invisibility of role models, limited senior buy‐in to strategic change, ableist assumptions, and disorganised infrastructure.^[27]^ An edited book theorising data and personal experiences of disabilities and chronic illnesses in higher education has recently been published and is freely available online (Ableism in Academia).^[28]^

## COVID‐19 Pandemic

“*Although the novel coronavirus affects every person and community, it does not do so equally. Instead, it has exposed and exacerbated existing inequalities and injustices*.” The UN Sustainable Development Goals Report, 2020^[29]^

The UN Sustainable Development Goals have been updated to highlight that disabled people are amongst those “*being hit hardest by the pandemic”* and that “*existing patterns of discrimination may be entrenched by the crisis*”.^[30]^ Disabled scientists have experienced significant and diverse challenges; here are just a few examples. Many have reported increased difficulties with accessing health and social care, long delays to reasonable adjustments, and variable levels of digital accessibility which makes living, learning, and working online more challenging.^[31]^ Scientists shielding for medical reasons have not been able to access labs and equipment necessary for their research and may also have felt isolated from colleagues and networks. Mask wearing is an important safety precaution but the necessity for their widespread use has had a substantial impact on scientists with hearing impairments.[^32^, ^33^] For those managing mental health conditions, the uncertainty, health and hygiene concerns, and isolation can be particularly challenging. Furthermore, frequent, and public discussion of herd immunity and underlying health conditions has made some feel required to assert that their disabled life is very much valued, rich, and important.[^34^, ^35^, ^36^] Others have felt forgotten about or side‐lined.

The pandemic has also had another effect, it has impacted disability reporting with many staff needing to share inforation about ‘underlying health conditions’ to line‐managers when negotiating arrangements for working at home or returning to face – face work. Will this increase in declaration of disability status continue and will inclusive and supportive working cultures develop as a result of increased visibility? It will be interesting to learn from the experiences of those who have chosen to declare during the pandemic.

There have also been some significant positives with regard to accessibility and inclusivity necessitated by the pandemic. Flexible and remote ways of working have been widely adopted, many scientific conferences and meetings have moved online (free or at a reduced fee), and increased use of video conferencing have reduced isolation. However, challenges remain for many disabled scientists as recently highlighted by Krystal Vasquez, including inaccurate captions and inaccessible presentations.^[37]^

## Looking Forward: Removing Barriers Faced by Disabled Scientists

When we transition into a post‐COVID‐19 world will the new normal include the continuation of some of the ways of working and studying which are inclusive and accessible? Can we harness tools, practices, and flexible ways of working to ensure that talented and creative scientists are not lost or held back due to a lack of accessibility or understanding? Will we also learn from the mistakes made and listen to the experiences of disabled scientists and students? Staff networks are an important resource for peer‐support and activism, and I have found being a member of my local network invaluable since beginning an academic career. Recently, Women in Supramolecular Chemistry (WISC) have announced a disability, chronic illness, and neurodivergency cluster in their network. The National Association of Disabled Staff Networks (NADSN) have called for more support for disabled scientists,^[38]^ and published a position paper reflecting on the diverse experience of the members including key recommendations for the future.^[31]^ This is a great starting point to learn from the experiences of disabled staff.

“*We strongly recommend that HEIs use this pandemic situation as an opportunity to ensure that any line management training, policies and procedures pertaining to managing Disabled workers (e.g. absence management, capability, carers, health, safety and wellbeing, personal and career development reviews, etc*.)*, are robustly equality impact assessed/analysed if they have not already. This will ensure they do not have a differential or adverse impact on certain groups, which are protected by law against discrimination.”* NADSN^[31]^

### Inclusive decision making: Ensure staff with disabilities are meaningfully involved in decision making

The slogan “Nothing about us, without us” is well known amongst disability activists and should be applied to all inclusion work attempting to address inequalities in access to science. This means disabled scientists should be consulted on all decisions which impact upon them and should be well represented amongst decision makers. There are practical considerations to help facilitate inclusion including ensuring meetings (including the technologies used) are accessible, they are scheduled thoughtfully to be inclusive to as wide a range of participants as possible, individuals should be able to contribute asynchronously as well as synchronously, and they need to be chaired in a considerate way to ensure all voices are listening to and valued. Disabled staff networks are key to this and should be consulted and supported.

### Remote working

Working remotely can be both enabling and isolating. The COVID‐19 pandemic has necessitated a widespread adoption of remote working for many of us and in many instances has shown that physical presence is not always necessary for productive scientific work. The ability to work from home flexibly can remove barriers for disabled scientists allowing them to organise their work in a way that suits them and reduce commuting and business travel. The option to work entirely from home can enable scientists to participate in learning and research who are house‐bound due to chronic conditions, such as chronic fatigue syndrome (CFS, also known as ME which stands for myalgic encephalomyelitis), or more recently due to a need to “shield” to minimise risk of COVID‐19 infection. As many of us can now attest to, working from home can also be isolating, and can be more difficult to connect meaningfully with colleagues. Staff working exclusively from home can miss out on networking and relationship opportunities, which can have negative effects on their morale, development, and progression. If returning to the office or lab, it will be important to connect with and reach out to those continuing to work remotely, ensuring that everyone has the opportunity to attend meetings and social events.

### Flexible working

Access to alternative working patterns (including job‐sharing) is a key recommendation of the recent JISC study into disability STEM data commissioned by the Royal Society, where the authors highlight that these arrangements should be available for senior roles in order to make these roles more appealing to scientists with disabilities.^[18]^ The ability to change working patterns, hours, and locations, allows people to arrange their work in the best way to meet their needs and can be particularly useful when living with fluctuating or unpredictable conditions. My own department encourages flexible working arrangements and supports and champions part‐time staff.^[39]^ These are inclusive policies which also help to encourage a healthy work‐life balance and benefit individuals with caring responsibilities.

### Developing inclusive cultures

We have expectations of what it is to be a scientist and what are (normal) ways of working as a scientist. The pressure to excel, to be perfect, to be competitive, whether internal or external, can prevent scientists from sharing their full selves in their workplaces. This can lead to individuals pushing themselves too hard, leading to negative impacts on their health and wellbeing. There has been great work highlighting the importance of allowing and encouraging people to be their true and whole selves at work amongst the LGBT+ community. This has included workplace environment studies,^[40]^ practical toolkits to improve inclusion,^[41]^ role‐models sharing their personal stories,[^42^, ^43^, ^44^] and opportunities to showcase the work of LGBT+ scientists.^[45]^ Research into disability declaration amongst STEM academics identified visible senior role models as a significant positive factor.^[23]^ It is important that scientists can talk openly and honestly about their lives with chronic conditions and mental ill health without fear of the judgements of others and particularly of those judgements extending to their abilities as scientists. Campaigns which encourage scientists to share their experiences of disability, such as the Voices of Academia mental health focussed project, can raise the voices of disabled scientists, build community, empathy, and awareness.

### How can we be allies?

Allies are vital in the creation of supportive environments where disabled staff can talk openly about their personal circumstances and where lived experience of disability is valued.

You could support your disabled colleagues by working as an ally, challenging ableist language and practices and reflecting on the fairness and accessibility of recruitment, performance management, or promotion procedures at your place of work or common in your industry.

So what could this look like?

- Consider what actions you can take to ensure that disabled people feel welcome and valued. Ask your employer whether they have disability inclusive policies, such as flexible working hours or part‐time work insurances.
- Find out if there is a disabled network at your place of work and offer your time as an ally. If you are a line manager, value when your supervisees undertake this type of service.
- If you hold a leadership position, ensure new lab spaces are born accessible and lobby for existing spaces to be renovated.

Overall, take time and space for empathy, compassion, and understanding. Many scientists do not disclose their personal circumstances and the majority of disabilities are invisible. I have learned so much from listening to students and staff with disabilities share their experiences, insights, and ideas. My key piece of advice would be to encourage you to start with reaching out to your colleagues and listening. Allies have an important role in enabling disabled staff and students to be fully involved in decision making and encouraging inclusive cultures.

## Key Recommendations

- Decision‐making: Ensure meaningful consultation with disabled staff and representation of disabled staff in committees and decision‐making groups.
- Allies must advocate for scientists from under‐represented groups to lessen the burden and risks of activism.
- Work with and support disabled staff networks.
- Web‐accessibility: Ensure online content including websites, learning management systems, and apps are accessible. At the minimum they should meet the EU Web Accessibility Directive by being perceivable, operable, understandable, and robust (For more information see the web accessibility initiative guide).
- Continue to offer flexible and remote working polices as recommended by the World Health Organisation.^[46]^
- Continue allowing virtual attendance of conferences and meetings and plan for inclusivity (see recommendations by Nic Flemming^[47]^ and Armstrong et al.^[48]^).

I would strongly encourage you to read the recommendations informed by extensive studies of the experiences of disabled STEM academics published by the Royal Society in collaboration with Jisc^[18]^ and the Careers, Research, and Advisory Centre,^[23]^ the recommendations of the NADSN,^[31]^ and those reported in the final chapter of Ableism in Academia.^[28]^

## Closing Remarks

“*I want my field not to underuse, misuse or neglect the human potential we all have for exploration and inquiry, and to trust that we all may contribute just as we are*.” Dr. Diaz‐Merced

The COVID‐19 pandemic has uniquely and considerably impacted on people with disabilities. Looking ahead, the “new normal” should be designed to address barriers faced by students and staff with disabilities. We need to be mindful of what new ways of working we keep, introduce, and say goodbye to. By ensuring we are inclusive of and supportive to as diverse a range of scientists as possible we can make sure that scientists represent the wider population, make space for new ideas, and ultimately new and exciting scientific developments.

Scientific institutions and workplaces must learn from the experiences of disabled scientists, champion and support disabled leaders, ensure the visibility of positive senior disabled role models, and adopt inclusive practices and policies. A culture change is required to ensure that disabled people are valued and recognised as scientists, innovators, and leaders. We need to support disabled scientists not just out of a moral inclusive obligation but also to avoid losing the immense talent of these individuals, leaving the scientific community much poorer.

Amongst the many difficulties faced during this pandemic there are also opportunities to remove barriers which prevent disabled scientists, including those with chronic illnesses, from thriving. Flexible and remote working practices, and technological solutions adopted during this time should continue to be available to allow scientists to carry out their research regardless of impairment and personal circumstances. Adopting flexible and compassionate approaches will benefit all scientists. The future will be reshaped by our experiences of and responses to the global COVID‐19 pandemic.

## Disclaimer

Science Voices are opinion articles written by scientists around the world and the views and opinions expressed in this article are those of the authors and not necessarily those of Wiley‐VCH.

## Conflict of interest

The authors declare no conflict of interest.

## Biographical Information

*Dr. Julia Sarju is a lecturer in Chemistry at the University of York, with interests in developing and encouraging adoption of inclusive teaching practices. She has lived experience of disability and supports students and staff with disabilities in her department*.